# Cocaine, amphetamine, or titin: Unraveling the genetic underpinnings of dilated cardiomyopathy

**DOI:** 10.1002/ccr3.9069

**Published:** 2024-06-11

**Authors:** Binay Kumar Panjiyar, Nikita Changlani, Clarisa Medina, Lisa R. Trevino

**Affiliations:** ^1^ GCSRT PGME, Harvard Medical School Boston Massachusetts USA; ^2^ Texas Tech University Health Sciences Center El Paso Paul L Foster School of Medicine El Paso Texas USA; ^3^ DHR Health Institute for Research and Development Edinburg Texas USA

**Keywords:** cardiology, dilated cardiomyopathy, heart failure, ischemic cardiomyopathy, titin mutation

## Abstract

**Key Clinical Message:**

An interesting case that shows the importance of identifying a pathogenic TTN gene mutation through genetic assessment in unexplained cardiomyopathy, especially with family history. This case highlights the need for genetic counseling and testing for at‐risk relatives, and advocates for personalized management considering both genetic and lifestyle factors.

**Abstract:**

This case report examines a 33‐year‐old Hispanic male with bipolar disorder, schizophrenia, and a history of substance use, presenting with acute respiratory failure and cardiac arrest. The patient's nonischemic dilated cardiomyopathy (DCM) highlights the critical role of genetic factors, particularly titin gene (TTN) mutations, in cardiomyopathy pathogenesis. Through genetic analysis, we explore the intersection of lifestyle factors and genetic predisposition in DCM, underscoring the importance of comprehensive genetic testing for accurate diagnosis and targeted therapy. This case contributes to the evolving understanding of DCM etiology, emphasizing the necessity of considering both environmental and genetic factors in clinical assessment and management.

## 1 INTRODUCTION

Nonischemic dilated cardiomyopathy (DCM) is characterized by ventricular dilation and impaired systolic function, not resulting from coronary artery disease or abnormal loading conditions.^1^ It has a diverse etiology, including genetic factors like titin (TTN) gene mutations. TTN, a crucial sarcomeric protein, maintains cardiomyocyte structural integrity and elasticity. Its mutations, particularly titin‐truncating variants (TTNtvs), are implicated in DCM, influencing cardiomyopathy's clinical course.^1^, ^2^

This case report aims to explore the complex interplay of genetic and environmental factors in DCM, with a focus on the role of TTNtvs. We present a case of a young male with DCM, examining the contributions of genetic predisposition and lifestyle factors, including substance abuse, to his cardiac pathology. The report underscores the importance of genetic testing in DCM diagnosis and management, especially in patients with a family history of cardiomyopathy.

## 2 CASE HISTORY/EXAMINATION

A 33‐year‐old Hispanic male with a history of bipolar disorder and schizophrenia was admitted to the emergency department (ED) with acute hypoxemic respiratory failure in 2022. Notably, he had a history of substance abuse, primarily cocaine, although he had been abstinent for approximately 4 months, continuing amphetamine use.

Upon arrival in the ED, the patient experienced cardiac arrest with pulseless electrical activity (PEA). Immediate and comprehensive resuscitative efforts, in line with Advanced Cardiovascular Life Support (ACLS) guidelines, were initiated. These included endotracheal intubation and intravenous epinephrine administration. Despite these interventions, return of spontaneous circulation was achieved after only 20 min.

The patient's post‐resuscitation period was marked by severe cardiogenic shock and a recurrence of cardiac arrest, again presenting with PEA. The combined efforts of the ED and intensive care unit (ICU) teams successfully resuscitated him. He was then admitted to the ICU and required multiple intravenous vasopressor drips and inotropic agents to maintain hemodynamic stability.

## 3 METHODS (DIFFERENTIAL DIAGNOSIS, INVESTIGATIONS, AND TREATMENT)

Cardiovascular assessment, including an echocardiogram performed immediately after the second cardiac arrest, revealed a dilated left ventricle with a severely reduced ejection fraction, initially estimated to be between 5% and 10%. A repeat echocardiogram 48 h later showed an improved ejection fraction of 20%–25% while on IV inotropic agent milrinone and multiple IV vasopressors. The initial EKG upon admission showed sinus tachycardia at 133 beats per minute, with no evidence of ST elevation or infarct pattern.

The patient's clinical course was further complicated by multisystem organ failure, encompassing anoxic encephalopathy and acute renal failure. Urine toxicology screening upon admission indicated positive results for amphetamines but negative for cocaine and other substances, aligning with the patient's history of substance abuse.

Significantly, the patient had a prior hospitalization in 2019 for an overdose and toxicity related to cocaine, amphetamines, and cannabinoids. His family history was notable for familial DCM and cardiac arrhythmias. The patient's father had a history of familial DCM, heart failure with reduced ejection fraction, necessitating implantable cardioverter‐defibrillator placement, electrophysiological ablation for atrial fibrillation, and a diagnosis of cardiomyopathy. Furthermore, two of the patient's paternal uncles had cardiomyopathy with advanced heart failure, and both underwent cardiac transplant procedures. Genetic testing revealed a TTN mutation in one of the patient's uncles.

## 4 CONCLUSION AND RESULTS (OUTCOME AND FOLLOW‐UP)

During his hospitalization, the patient's condition remained critical, necessitating prolonged intubation, mechanical ventilation, and hemodialysis for acute renal failure. His overall hemodynamic status remained tenuous, and a neurology evaluation, including EEG, was consistent with brain death. The family ultimately requested the withdrawal of life support in 2022.

Genetic and metabolic profiling performed on the patient (collection date: 2022) revealed a significant finding. A heterozygous c.67279C>T (p. Arg22427Ter) pathogenic variant in the TTN (NM_001267550.1) gene was detected in the patient's sample, suggesting a genetic predisposition associated with the TTN gene. Therefore, substances, like cocaine and amphetamines which have known cardiotoxic effects, should not be made the only culprit in cases where family history is positive for cardiomyopathy. This might have played a role in the delayed diagnosis of truncated TTN‐induced cardiomyopathy.

This case presents a complex clinical scenario of a young adult with psychiatric illness, substance abuse, familial DCM, and a tragic course marked by acute respiratory failure, cardiac arrest, and severe cardiogenic shock. The discovery of a TTN gene pathogenic variant in the patient underscores the importance of considering genetic factors in cases of unexplained cardiomyopathy, especially in the context of a family history of cardiac disease. Genetic counseling and further genetic testing for at‐risk family members are recommended to better understand and manage the genetic predisposition identified in this case.

## 5 DISCUSSION

This case underscores the intricate relationship between genetic factors and DCM, particularly the role of TTNtvs. TTN, the largest known human protein, is crucial in maintaining cardiac structure and function. Truncations in the TTN gene are a significant cause of DCM, as evidenced by Herman et al. (2012).^1^ These TTNtvs, while not uniformly leading to DCM, significantly increase the risk.

The pathophysiology of TTNtvs in DCM involves a disrupted sarcomere structure, which impairs cardiac muscle contractility and leads to heart failure.^1^ Research by Schafer et al. (2015)^3^ indicates that TTNtvs are prevalent in both familial and sporadic cases of DCM, highlighting their importance in the disease's genetic architecture.^3^ Furthermore, the haploinsufficiency model suggests that a loss of one functional allele of TTN can lead to insufficient protein production, contributing to DCM pathogenesis.

However, not all TTNtvs result in DCM, indicating a variable penetrance and expressivity. This variability, as explored by Roberts et al. (2015)^4^ is influenced by additional genetic modifiers and environmental factors.^4^ Consequently, the clinical presentation of DCM in individuals with TTNtvs can range from asymptomatic to severe heart failure, underscoring the complexity of predicting disease progression and the necessity for personalized approaches to patient care.

In the present case, the patient's history of substance abuse complicates the clinical picture. Amphetamines and cocaine are known to have cardiotoxic effects, potentially exacerbating the underlying genetic predisposition to DCM. Cocaine directly damages the heart, causing myofibrils to break down, interstitial fibrosis, myocardial dilatation, and heart failure. Amphetamine causes increased heart rate, contractility, and afterload leading to signs of cardiac ischemia and dysfunction. This emphasizes the need for a comprehensive approach to diagnosis and management, considering both genetic and environmental factors.

The discovery of TTNtvs in our patient not only guided the clinical management but also highlighted the importance of genetic counseling for at‐risk family members.^3^, ^5^ Genetic testing for TTNtvs has become a vital tool in the diagnosis and management of DCM, aiding in the identification of familial cases and guiding therapeutic decisions.

In conclusion, this case report illustrates the multifaceted nature of DCM, where genetic predisposition interplays with environmental factors, such as substance abuse, to manifest the disease. It reinforces the need for genetic evaluation in unexplained cardiomyopathies and personalized patient care, considering both genetic background and lifestyle factors.

## AUTHOR CONTRIBUTIONS

**Binay Kumar Panjiyar:** Conceptualization; investigation; methodology; project administration; writing – original draft; writing – review and editing. **Nikita Changlani:** Conceptualization; formal analysis; investigation; methodology; validation; writing – review and editing. **Clarisa Medina:** Formal analysis; investigation; methodology; project administration; writing – review and editing. **Lisa R. Trevino:** Methodology; project administration; writing – review and editing.

## FUNDING INFORMATION

No funding has been received from any sources.

## ETHICS STATEMENT

This study was conducted in accordance with the ethical standards of our institution and with the 1964 Helsinki declaration and its later amendments or comparable ethical standards.

## CONSENT

Written informed consent was obtained from the patient to publish this report in accordance with the journal's patient consent policy.

## Data Availability

Data sharing is not applicable as no new data were generated, or the article describes entirely theoretical research.

## References

[1] Herman DS , Lam L , Taylor MR , et al. Truncations of titin cause dilated cardiomyopathy. N Engl J Med. 2012;366(7):619‐628. 22335739 10.1056/NEJMoa1110186 PMC3660031

[2] Clark KA , McElhinny AS , Beckerle MC , Gregorio CC . Striated muscle cytoarchitecture: an intricate web of form and function. Annu Rev Cell Dev Biol. 2002;18:637‐706. 12142273 10.1146/annurev.cellbio.18.012502.105840

[3] Schafer S , de Marvao A , Adami E , et al. Titin‐truncating variants affect heart function in disease cohorts and the general population. Nat Genet. 2015;49(1):46‐53. 10.1038/ng.3719 PMC5201198 27869827

[4] Roberts AM , Ware JS , Herman DS , et al. Integrated allelic transcriptional and phenomic dissection of the cardiac effects of titin truncations in health and disease. Sci Transl Med. 2015;7(270):270ra6. 10.1126/scitranslmed.3010134 PMC4560092 25589632

[5] Hershberger RE , Givertz MM , Ho CY , et al. Genetic evaluation of cardiomyopathy: a clinical practice resource of the American College of Medical Genetics and Genomics (ACMG). Genet Med. 2018;20(9):899‐909. 29904160 10.1038/s41436-018-0039-z

